# Lipid-lowering drugs affect lung cancer risk via sphingolipid metabolism: a drug-target Mendelian randomization study

**DOI:** 10.3389/fgene.2023.1269291

**Published:** 2023-11-16

**Authors:** Honglin Li, Lei Zhang, Feiran Yang, Xiaoteng Feng, Rong Fu, Ruohan Zhao, Xiurong Li, Huijie Li

**Affiliations:** ^1^ First Clinical College, Shandong University of Traditional Chinese Medicine, Jinan, Shandong, China; ^2^ Department of Oncology, Affiliated Hospital of Shandong University of Traditional Chinese Medicine, Jinan, Shandong, China; ^3^ Department of Cardiology, Longhua Hospital, Shanghai University of Traditional Chinese Medicine, Shanghai, China; ^4^ Department of Oncology, Longhua Hospital Affiliated to Shanghai University of Traditional Chinese Medicine, Shanghai, China

**Keywords:** lipid-lowering drug, ceramide, ceramidase, sphingosine-1-phosphate, sphingolipid metabolism, lung cancer, Mendelian randomization study

## Abstract

**Background:** The causal relationship between lipid-lowering drug (LLD) use and lung cancer risk is controversial, and the role of sphingolipid metabolism in this effect remains unclear.

**Methods:** Genome-wide association study data on low-density lipoprotein (LDL), apolipoprotein B (ApoB), and triglycerides (TG) were used to develop genetic instrumental variables (IVs) for LLDs. Two-step Mendelian randomization analyses were performed to examine the causal relationship between LLDs and lung cancer risk. The effects of ceramide, sphingosine-1-phosphate (S1P), and ceramidases on lung cancer risk were explored, and the proportions of the effects of LLDs on lung cancer risk mediated by sphingolipid metabolism were calculated.

**Results:** *APOB* inhibition decreased the lung cancer risk in ever-smokers via ApoB (odds ratio [OR] 0.81, 95% confidence interval [CI] 0.70–0.92, *p* = 0.010), LDL (OR 0.82, 95% CI 0.71–0.96, *p* = 0.040), and TG (OR 0.63, 95% CI 0.46–0.83, *p* = 0.015) reduction by 1 standard deviation (SD), decreased small-cell lung cancer (SCLC) risk via LDL reduction by 1 SD (OR 0.71, 95% CI 0.56–0.90, *p* = 0.016), and decreased the plasma ceramide level and increased the neutral ceramidase level. *APOC3* inhibition decreased the lung adenocarcinoma (LUAD) risk (OR 0.60, 95% CI 0.43–0.84, *p* = 0.039) but increased SCLC risk (OR 2.18, 95% CI 1.17–4.09, *p* = 0.029) via ApoB reduction by 1 SD. *HMGCR* inhibition increased SCLC risk via ApoB reduction by 1 SD (OR 3.04, 95% CI 1.38–6.70, *p* = 0.014). The *LPL* agonist decreased SCLC risk via ApoB (OR 0.20, 95% CI 0.07–0.58, *p* = 0.012) and TG reduction (OR 0.58, 95% CI 0.43–0.77, *p* = 0.003) while increased the plasma S1P level. *PCSK9* inhibition decreased the ceramide level. Neutral ceramidase mediated 8.1% and 9.5% of the reduced lung cancer risk in ever-smokers via ApoB and TG reduction by *APOB* inhibition, respectively, and mediated 8.7% of the reduced LUAD risk via ApoB reduction by *APOC3* inhibition.

**Conclusion:** We elucidated the intricate interplay between LLDs, sphingolipid metabolites, and lung cancer risk. Associations of *APOB*, *APOC3*, and *HMGCR* inhibition and *LPL* agonist with distinct lung cancer risks underscore the multifaceted nature of these relationships. The observed mediation effects highlight the considerable influence of neutral ceramidase on the lung cancer risk reduction achieved by *APOB* and *APOC3* inhibition.

## 1 Introduction

The incidence and mortality rates of lung cancer have consistently increased in accordance with global patterns of malignant tumor (Sharma, 2022). Despite continuous advancements and refinements in lung cancer treatment, the pursuit of effective prevention methods to mitigate the risk of developing this disease remains paramount (Kordiak et al., 2022). Observational studies have suggested a potential association between statin use and a reduced risk of lung cancer (Khurana et al., 2007; Yang et al., 2015; Kwon et al., 2019). However, a case–control study conducted within the UK population reported an increased risk of lung cancer associated with long-term statin exposure (Vinogradova et al., 2011). Furthermore, recent Mendelian randomization (MR) studies have challenged the notion of a causal relationship (Carter et al., 2020; Min et al., 2023). Investigations of lipidomic profiles revealed that the use of statins, PCSK9 inhibitors, and fenofibrate, instead of NPC1L1 inhibitors, results in a reduction in plasma ceramide and sphingomyelin levels (Ng et al., 2014; Tarasov et al., 2014; Ng et al., 2015; Croyal et al., 2018). Nonetheless, findings regarding the impact of statins on plasma sphingosine-1-phosphate (S1P) levels have exhibited inconsistency (Egom et al., 2013; Therond and Chapman, 2022). Preclinical investigations have elucidated the significant role of sphingolipid metabolism in the pathogenesis of lung cancer (Ogretmen, 2018; Meng et al., 2021; Tang et al., 2023), although the precise relationship remains unclear (Lin et al., 2022). Among the sphingolipids, ceramide and S1P have garnered particular attention within lung cancer studies (Goldkorn et al., 2013). Notably, ceramidases play a pivotal role in the conversion of ceramide to S1P (Pyne and Pyne, 2010). While a case–control study has uncovered associations between higher concentrations of plasma sphingosine-1-phosphate and ceramides and the increasing risk of lung cancer, these findings contradict the preclinical results that suggest that ceramides promote apoptosis in lung cancer cells (Ogretmen and Hannun, 2004; Carpinteiro et al., 2008; Alberg et al., 2013). Evidence on how lipid-lowering drugs (LLDs) may influence the risk of lung cancer by modulating sphingolipid metabolites remains elusive, along with the potential underlying mechanisms.

To explore the intricate relationships among LLDs, sphingolipid metabolites (including plasma ceramide, ceramidase, and S1P), and the risk of lung cancer, we employed a drug-target MR analysis (Holmes et al., 2021) to address a critical knowledge gap and provide valuable insights into potential chemoprevention in lung cancer.

## 2 Materials and methods

### 2.1 Study design

The results obtained from MR analysis, using data from genome-wide association studies (GWAS) to analyze the effects of exposure factors, closely resemble the findings of randomized controlled trials. This resemblance can be attributed to the random allocation of genetic variants during meiosis (Yarmolinsky et al., 2022). The expression and functionality of drug targets can be profoundly affected by genetic variations. Moreover, the impact of drugs can be anticipated through the genetic variability present in the genes encoding their protein targets (Chauquet et al., 2021). Similar to the previous study, instrumental variables (IVs) for LLDs were extracted from GWAS summary statistics related to low-density lipoprotein (LDL), apolipoprotein B (ApoB), and triglycerides (TG), enabling an analysis of the causal relationship between LLD exposure and the risk of developing lung cancer (Williams et al., 2020; Xiao et al., 2023). To clarify the mediating role of sphingolipid metabolites, we employed a two-step MR analysis. This involves utilizing the outcome variable from the initial MR analysis as the exposure variable for the subsequent MR analysis (Adams and Boutwell, 2021; Xu et al., 2022). Our analysis investigates the impact of LLDs on plasma sphingolipid metabolite (ceramide, S1P, and ceramidase) levels, alongside establishing a causal link between these sphingolipid metabolites and lung cancer risk. Additionally, we quantify the mediating influence of sphingolipid metabolites on the association between LLDs and lung cancer risk.

### 2.2 Genetic variant selection

Information about chromosomal and gene loci for the eight LLD targets (Williams et al., 2020), namely, *ANGPTL3*, *APOB*, *APOC3*, *HMGCR*, *LPL*, *NPC1L1*, *PCSK9,* and *PPARA*, was obtained from the National Center for Biotechnology Information gene database (https://www.ncbi.nlm.nih.gov/gene) (see Table 1 and Supplementary Table S1 for details).

**TABLE 1:** Information on lipid-lowering drug targets included in the studies.

Examples of drugs/class	Target	Gene encoding target	HGNC ID
Evinacumab	Angiopoietin‐like protein 3 (ANGPTL3)	*ANGPTL3*	491
Mipomersen	Apo‐B 100 messenger RNA (ApoB‐100)	*APOB*	603
Volanesorsen	Apolipoprotein C‐3 (ApoC3)	*APOC3*	610
Statins	HMG‐CoA reductase (HMGCR)	*HMGCR*	5006
Alipogene tiparvovec	Lipoprotein lipase (LPL)	*LPL*	6677
Ezetimibe	Niemann–Pick C1‐like protein 1 (NPC1L1)	*NPC1L1*	7897
Evolocumab	Proprotein convertase subtilisin/kexin type 9 (PCSK9)	*PCSK9*	20001
Fibrates	Peroxisome proliferator‐activated receptor alpha (PPARα)	*PPARA*	9232

HGNC, HUGO Gene Nomenclature Committee.

We accessed the Medical Research Council Integrative Epidemiology Unit (IEU) OpenGWAS database (https://gwas.mrcieu.ac.uk/) and obtained GWAS summary statistics for LDL (ieu-a-300, *n* = 173,082), TG (ieu-a-302, *n* = 177,861), and ApoB (ieu-b-108, *n* = 439,214). These lipid traits were selected as they represent downstream substances influenced by the effectiveness of LLDs (Williams et al., 2020).

To generate IVs for LLDs, we used the LLD target genes, involving four targets of drugs for decreasing LDL (*APOB*, *HMGCR*, *NPC1L1*, and *PCSK9*), six targets of drugs for decreasing ApoB (*ANGPTL3*, *APOB*, *APOC3*, *LPL*, *PPARA*, and *PCSK9*), and five targets of drugs for decreasing TG (*ANGPTL3*, *APOB*, *APOC3*, *PPARA*, and *LPL*) (Xiao et al., 2023). We confined the single-nucleotide polymorphisms (SNPs) to those located within a 100-kb range surrounding the LLD target genes that exhibited genome-wide significance in their association with the lipid traits (*p* < 5.0 × 10^−8^). To maximize the IV strength for each LLD target, SNPs were allowed to be in weak linkage disequilibrium (*r* ^2^ < 0.30, window size = 10,000 kb) with each other (Xie et al., 2023). Given the absence of any genetic variation in *PPARA* during the selection process, it has been excluded from subsequent evaluations (Li et al., 2023).

IV strength was assessed using the F-statistic, with values > 10 indicating non-weak IVs (Duan et al., 2021). Additionally, for a positive control analysis, GWAS summary statistics for coronary heart disease (CHD) obtained from CARDIoGRAM (ieu-a-8, *n* = 86,995) were utilized as the outcome measure (Huang et al., 2021).

### 2.3 Sources of GWAS summary statistics for ceramidases and lung cancer

GWAS summary statistics for plasma ceramide (GCST90025189, *n* = 6,057) and S1P levels (GCST90199657, *n* = 8,246) were obtained from GWAS Catalog (Cadby et al., 2022; Chen et al., 2023). Ceramidases are classified into acid, neutral, and alkaline ceramidases based on their pH characteristics. However, in the available datasets, there were only GWAS summary statistics for acid and neutral ceramidases (prot-a-178 and prot-a-179, *n* = 3,301) from the IEU OpenGWAS database (Sun et al., 2018). The GWAS summary statistics for lung cancer were sourced from the publicly available dataset published by McKay et al. (2017), which included lung cancer cases (*n* = 85,716) as well as subgroups of ever-smokers (*n* = 40,187), never-smokers (*n* = 9,859), lung squamous cell carcinoma (LUSC) (*n* = 63,053), lung adenocarcinoma (LUAD) (*n* = 66,756), and small-cell lung cancer (SCLC) (*n* = 24,108). Table 2 lists the demographic characteristics of detailed lung cancer GWAS summary statistics.

**TABLE 2:** Demographic characteristics of GWAS statistics for lung cancer.

Subgroup	No. of patients	No. of controls	No. of sum
Overall lung cancer	29,266	56,450	85,716
Age			
<=50	3,112	6,032	9,144
>50	23,025	44,075	67,100
Sex			
Male	18,208	27,178	45,386
Female	11,059	24,069	35,128
Smoking status			
Never	2,355	7,504	9,859
Ever	23,223	16,964	40,187
Former	9,037	8,554	17,591
Current	13,356	7,477	20,833
Histology			
LUAD	11,273	55,483	66,756
LUSC	7,426	55,627	63,053
SCLC	2,664	21,444	24,108

LUAD, lung adenocarcinoma; LUSC, lung squamous cell carcinoma; SCLC, small-cell lung cancer.

### 2.4 Statistical analysis

MR analysis for the effects of LLDs on the risk of lung cancer and sphingolipid metabolite levels was calculated. The clumping procedure (*r* ^2^ < 0.001, window size = 10,000 kb) was performed to remove linkage disequilibrium, and SNPs that exhibited genome-wide significance (*p* < 5.0 × 10^−8^) were selected as IVs for ceramide, S1P, and acid/neutral ceramidases (Fang et al., 2023) (Supplementary Table S3).

When only one SNP was available as the IV, we utilized the Wald ratio method. Otherwise, we employed five methods: inverse variance-weighted (IVW), MR-Egger, simple mode, weighted median, and weighted mode. A reliable causal relationship was defined as follows: estimates from the IVW method or the Wald ratio method were statistically significant (*p* < 0.05) and were consistent with the direction of causal estimates computed by the MR-Egger method (Wang et al.).

To explore the mediating effect of sphingolipid metabolites regarding the effect of LLDs on lung cancer risk, a two-step MR analysis was performed. The proportion mediated by sphingolipid metabolites was calculated using the following formula (Chen et al., 2022; Zhao et al., 2022): $Ε\left(\%\right)=\frac{\Sigma_{k=1}^{k}\beta 1\times \beta 2k}{\Sigma_{k=1}^{k}\beta 3+\beta 1\times \beta 2k}$ , where β1 represents the estimated effect of LLDs on sphingolipid metabolites, β2 represents the estimated effect of sphingolipid metabolites on lung cancer, and β3 represents the estimated direct effect of LLDs on lung cancer. The product of β1 and β2 is considered the indirect effect, while (β3 + β1 × β2) represents the total effect. The proportion is calculated when β1, β2, and β3 exhibit statistical significance, as this quantifies the proportion of mediation by sphingolipid metabolites within the total effect (Chen et al., 2022). To account for multiple statistical tests and reduce false-positive results, we controlled the false discovery rate (FDR) using the Benjamini–Hochberg method. Adjusted *p* < 0.05 was considered statistically significant (Liu et al., 2023).

To assess the sensitivity of the MR results, we conducted MR-Egger intercept tests and calculated Cochran’s Q statistic to evaluate pleiotropy and heterogeneity. A significance level of *p* < 0.05 was applied to determine statistical significance. All MR analyses were performed using the “*TwoSampleMR*” package (version 0.5.6) in R software (version 4.1.1), and forest plots were generated using the “*forestploter*” package.

## 3 Results

### 3.1 Positive control analysis

**FIGURE 1:**
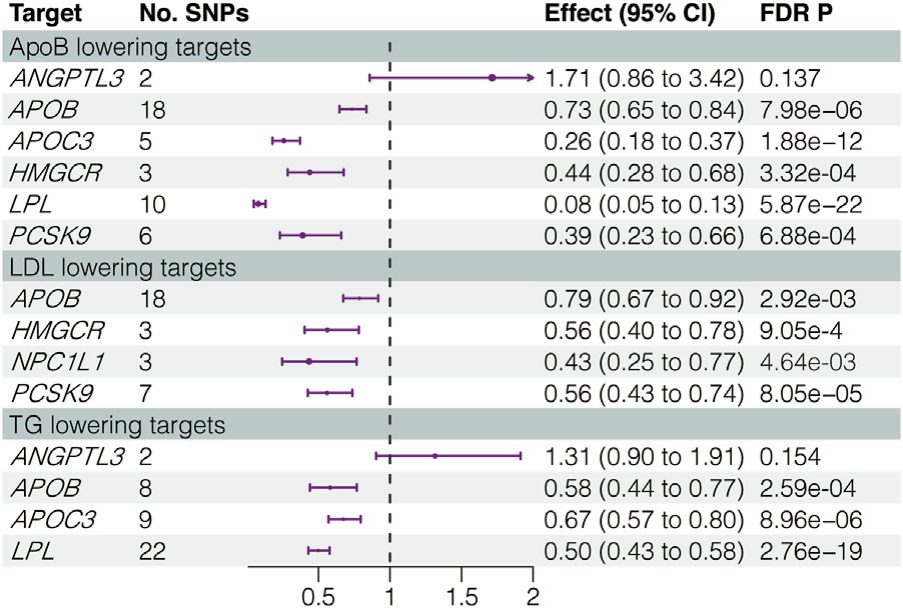
Results of positive control analysis. LDL, low-density lipoprotein; ApoB, apolipoprotein B; TG, triglycerides; SNPs, single-nucleotide polymorphisms; FDR, false discovery rate.

We investigated the causal associations between the IVs for inhibiting the LLD targets and CHD. Notably, the effect of *ANGPTL3* inhibition, whether via ApoB or TG reduction, did not show statistically significant results in the positive control analysis. Consequently, *ANGPTL3* inhibition was excluded from the subsequent MR analysis. The inhibition of the remaining six LLD targets exhibited a significant causal association, with a reduced risk of CHD. Figure 1 and Supplementary Table S4 show detailed results, illustrating the magnitude and significance of these associations.

### 3.2 The effects of LLDs on lung cancer risk

**FIGURE 2:**
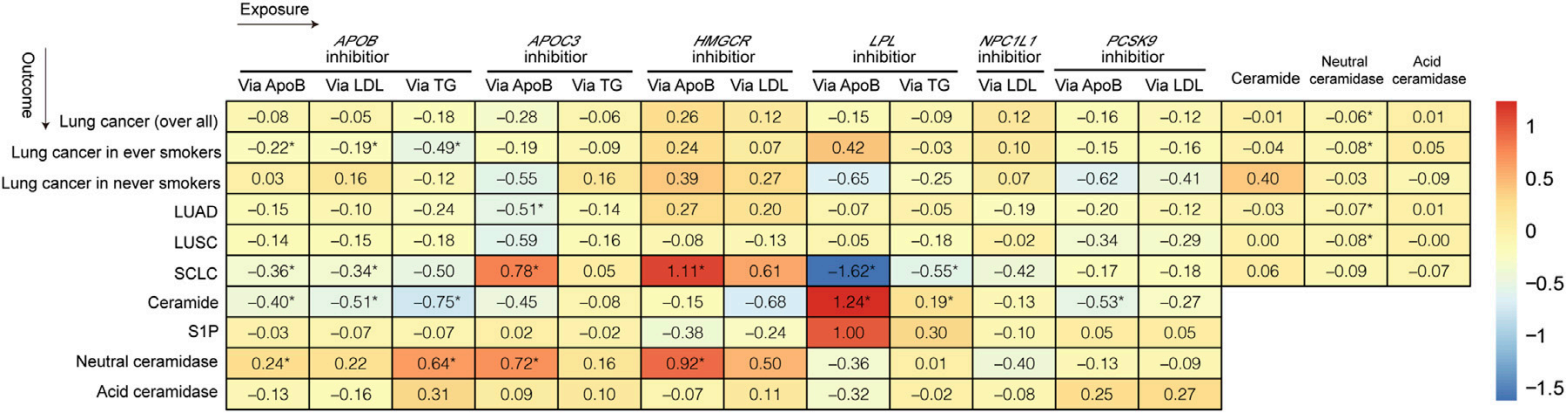
Heatmap of the causal relationship between LLDs, lung cancer, and ceramidases.

**FIGURE 3:**
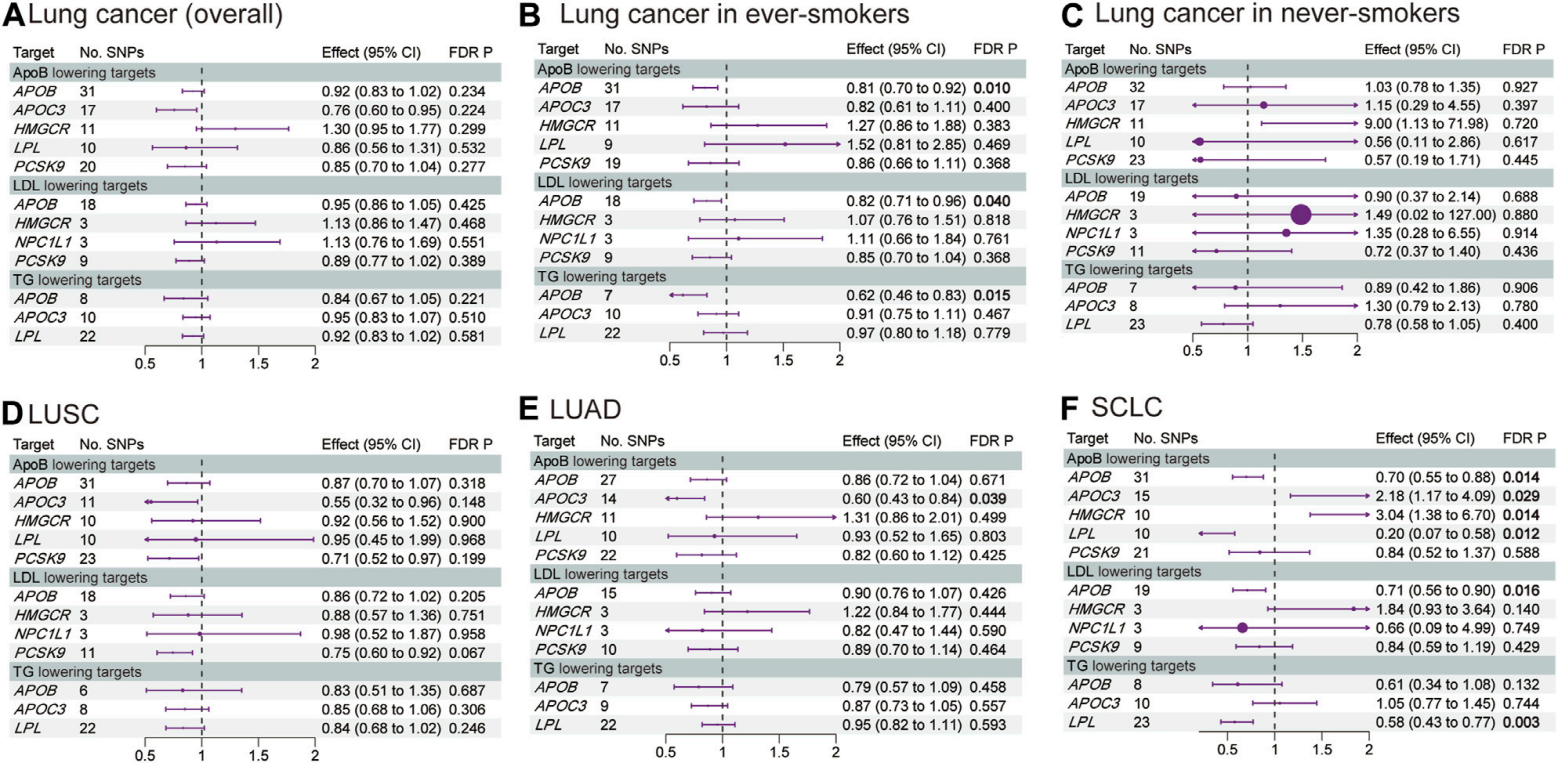
Estimated effects of LLD on the risk of **(A)** lung cancer (overall); **(B)** lung cancer in ever-smokers; **(C)** lung cancer in never-smokers; **(D)** LUSC; **(E)** LUAD; and **(F)** SCLC. FDR-adjusted *p*-values with statistical significance (<0.05) are shown in bold. LDL, low-density lipoprotein; ApoB, apolipoprotein B; TG, triglycerides; LUAD, lung adenocarcinoma; LUSC, lung squamous cell carcinoma; SCLC, small-cell lung cancer; FDR, false discovery rate.

Figures 2, 3 and Supplementary Tables S5–S10 show the results of our MR analysis, estimating the effects of LLDs on the risk of lung cancer (overall) and in five subgroups (lung cancer in ever-smokers, lung cancer in never-smokers, LUSC, LUAD, and SCLC).

*APOB* inhibition significantly decreased the risk of lung cancer in ever-smokers [odds ratio (OR) 0.81, 95% confidence interval (CI) 0.70–0.92, *p* = 0.01] and SCLC (OR 0.70, 95% CI 0.55–0.88, *p* = 0.014) via ApoB reduction by 1 standard deviation (SD). Additionally, *APOB* inhibition decreased the risk of lung cancer in ever-smokers via LDL (OR 0.82, 95% CI 0.71–0.96, *p* = 0.040) and TG (OR 0.62, 95% CI 0.46–0.83, *p* = 0.015) reduction by 1 SD. Furthermore, *APOB* inhibition decreased the risk of SCLC via LDL reduction (OR 0.71, 95% CI 0.56–0.90, *p* = 0.016).

*APOC3* inhibition decreased the risk of LUAD via ApoB reduction by 1 SD (OR 0.60, 95% CI 0.43–0.84, *p* = 0.039). However, it increased the risk of SCLC via ApoB reduction (OR 2.18, 95% CI 1.17–4.09, *p* = 0.029).

Interestingly, *HMGCR* inhibition increased the risk of SCLC via ApoB reduction by 1 SD (OR 3.04, 95% CI 1.38–6.70, *p* = 0.014). On the other hand, the *LPL* agonist decreased the risk of SCLC via ApoB (OR 0.20, 95% CI 0.07–0.58, *p* = 0.012) and TG (OR 0.58, 95% CI 0.43–0.77, *p* = 0.003) reduction by 1 SD. The MR analysis conducted exhibited neither pleiotropy nor heterogeneity.

### 3.3 The effects of LLDs on ceramide, S1P, and ceramidases

**FIGURE 4:**
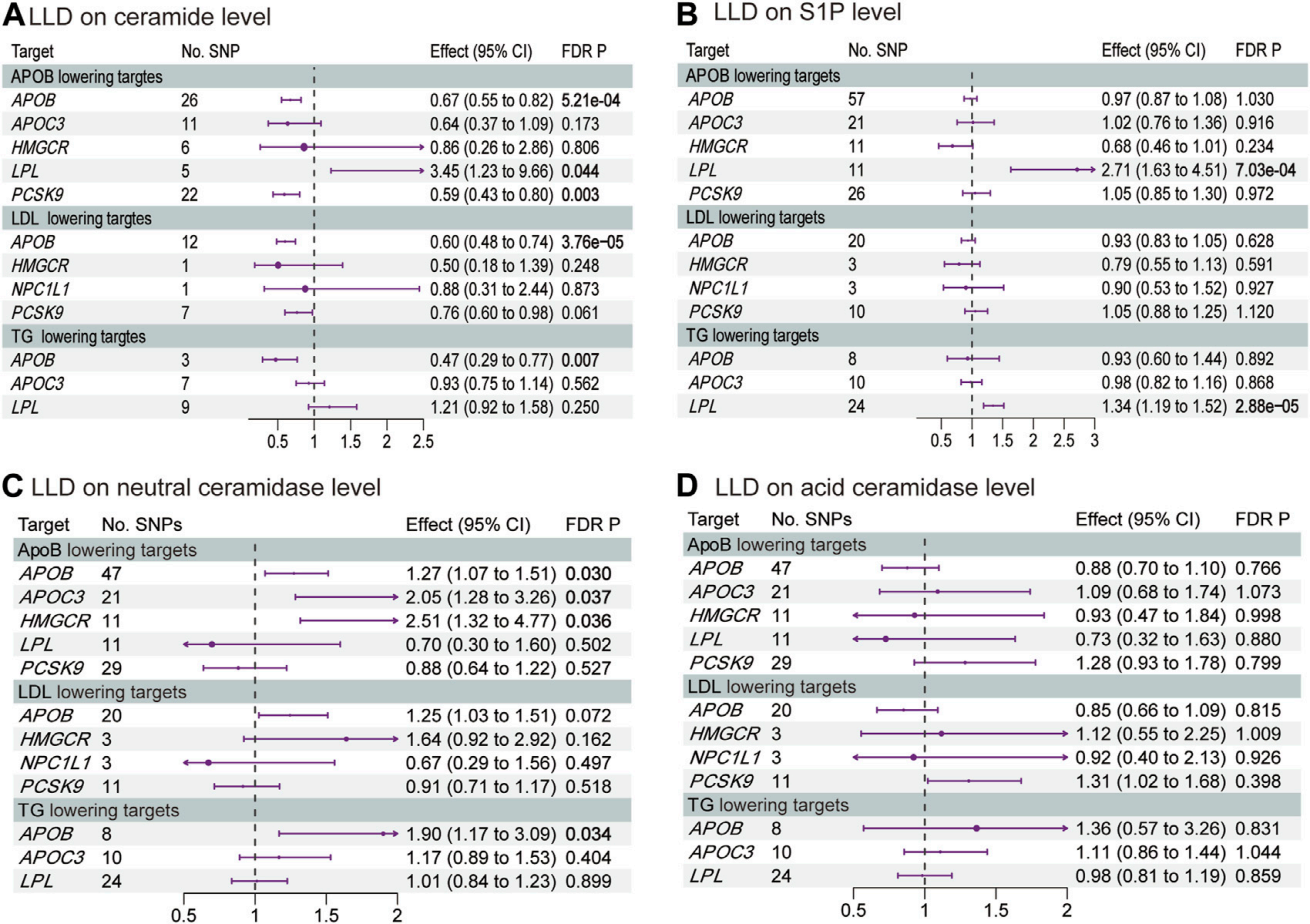
Estimated effects of LLD on plasma **(A)** ceramide; **(B)** S1P; **(C)** neutral ceramidase; and **(D)** acid ceramidase levels. FDR-adjusted *p*-values with statistical significance (<0.05) are shown in bold. LDL, low-density lipoprotein; ApoB, apolipoprotein B; TG, triglycerides; FDR, false discovery rate; S1P, sphingosine-1-phosphate.

Figure 4 and Supplementary Tables S11–S14 show the results of MR analysis, estimating the effects of LLDs on plasma ceramide, S1P, and ceramidases levels. *APOB* inhibition decreased the plasma ceramide level via ApoB (OR 0.67, 95% CI 0.55–0.82, *p* = 5.21 × 10^−4^), LDL (OR 0.60, 95% CI 0.48–0.74, *p* = 3.76 × 10^−5^), and TG (OR 0.47, 95% CI 0.29–0.77, *p* = 0.007) reduction by 1 SD. Similarly, *PCSK9* inhibition decreased the plasma ceramide level via ApoB (OR 0.59, 95% CI 0.43–0.80, *p* = 0.003) and LDL (OR 0.76, 95% CI 0.60–0.98, *p* = 0.061) reduction by 1 SD. Conversely, the *LPL* agonist increased the plasma ceramide level via ApoB (OR 3.45, 95% CI 1.23–9.66, *p* = 0.044) reduction by 1 SD and increased the plasma S1P level via ApoB (OR 2.71, 95% CI 1.63–4.51, *p* = 7.03 × 10^−4^) and TG (OR 1.34, 95% CI 1.19–1.52, *p* = 2.88 × 10^−5^) reduction by 1 SD. Interestingly, *APOB* inhibition increased the plasma neutral ceramidase level via ApoB (OR 1.27, 95% CI 1.07–1.51, *p* = 0.03) and TG (OR 1.90, 95% CI 1.17–3.09, *p* = 0.034) reduction by 1 SD. Although *APOB* inhibition increased the plasma neutral ceramidase level via LDL reduction, it did not reach statistical significance after correction (OR 1.25, 95% CI 1.03–1.51, *p* = 0.072). However, LLDs did not significantly impact plasma acid ceramidase levels. The MR analysis exhibited neither pleiotropy nor heterogeneity.

### 3.4 The effects of sphingolipid metabolites on lung cancer risk

**FIGURE 5:**
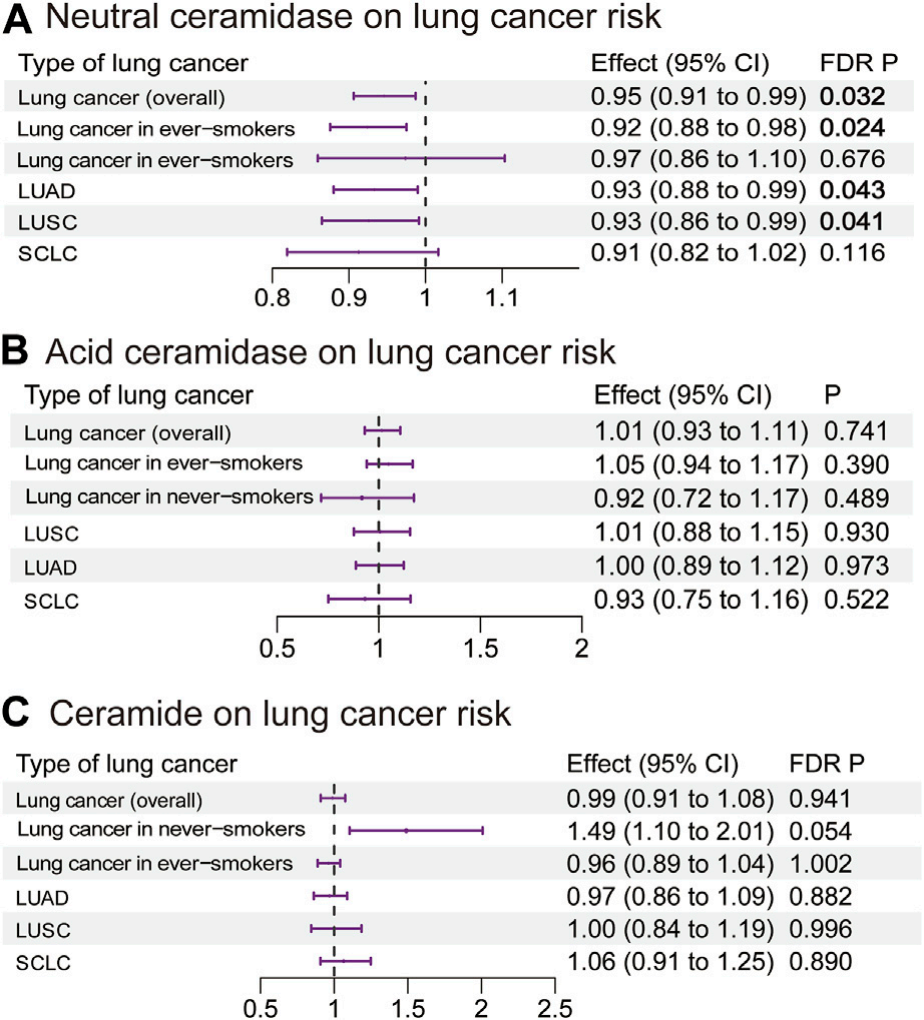
Estimated effects of plasma **(A)** neutral ceramidase; **(B)** acid ceramidase; and **(C)** ceramide levels on lung cancer risk. FDR-adjusted *p*-values with statistical significance (<0.05) are shown in bold. LUAD, lung adenocarcinoma; LUSC, lung squamous cell carcinoma; SCLC, small-cell lung cancer; FDR, false discovery rate.

Our MR analysis revealed that an increase in the plasma neutral ceramidase level by 1 SD decreased the risk of overall lung cancer (OR 0.95, 95% CI 0.91–0.99, *p* = 0.032), lung cancer in ever-smokers (OR 0.92, 95% CI 0.88–0.98, *p* = 0.024), LUAD (OR 0.93, 95% CI 0.88–0.99, *p* = 0.043), and LUSC (OR 0.93, 95% CI 0.86–0.99, *p* = 0.041) (Figure 5A; Supplementary Table S15). However, there was no evidence of a causal relationship between the plasma acid ceramidase level and risk of lung cancer (Figure 5B; Supplementary Table S16). The plasma ceramide levels exhibited a positive correlation with heightened lung cancer risk in never-smokers; however, this relationship did not achieve statistical significance following correction (OR 1.49, 95% CI 1.10–2.01, *p* = 0.054) (Figure 5C; Supplementary Table S17). As the IVs of S1P did not intersect with the SNPs present in the GWAS data for lung cancer, the execution of this MR analysis was rendered unfeasible. The MR analysis exhibited neither pleiotropy nor heterogeneity.

### 3.5 Analysis of the mediating effects of sphingolipid metabolites

**FIGURE 6:**
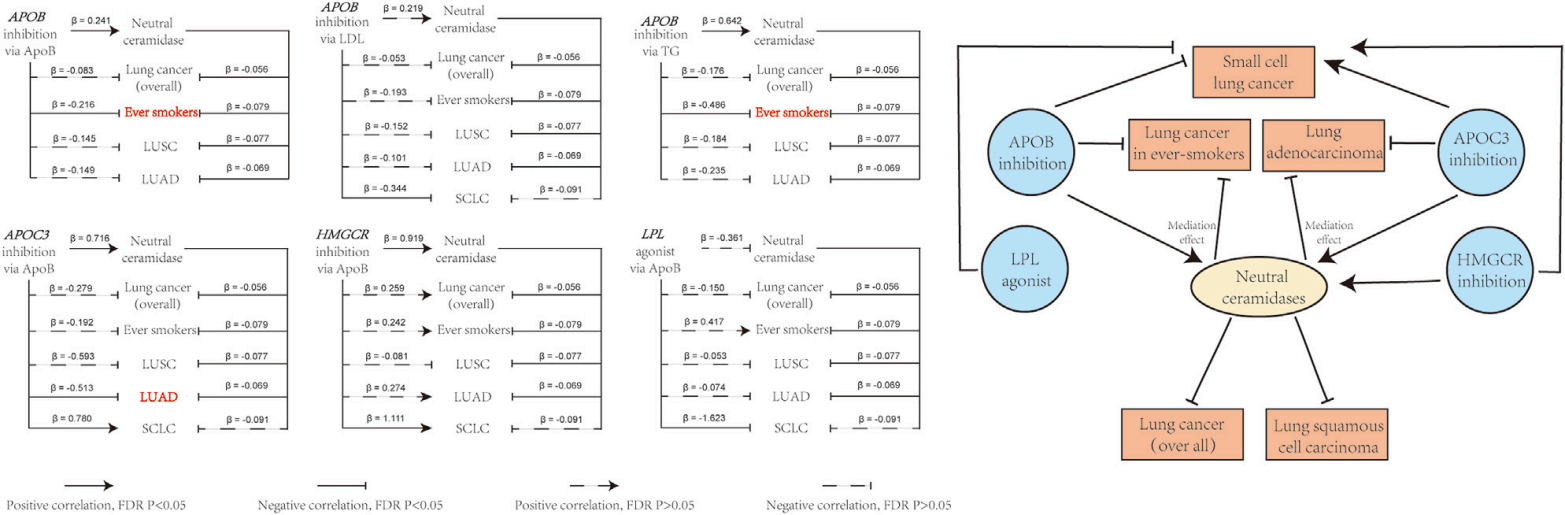
Overview of the direct and indirect effects of LLD on lung cancer risk, and the effects of neutral ceramidase. The effects of LLD on lung cancer risk involving mediating effects of neutral ceramidase are highlighted in red. LDL, low-density lipoprotein; ApoB, apolipoprotein B; TG, triglycerides.

The aforementioned results suggested that neutral ceramidase plays a mediating role in the effect of LLDs on lung cancer risk. Table 3 shows the proportions mediated by neutral ceramidase regarding the negative effect of *APOB* inhibition on lung cancer risk in smokers via ApoB and TG reduction (8.1% and 9.5%, respectively) and the negative effect of *APOC3* inhibition on LUAD risk via ApoB reduction (8.7%). Figure 6 illustrates schematic diagrams of the direct and indirect effects of LLDs on lung cancer and the mediation by neutral ceramidase.

**TABLE 3:** Proportions mediated by neutral ceramidase regarding the effects of LLD on lung cancer risk.

LLD target	Lung cancer subgroup	β 1	β 2	β3	Proportion (%)
*APOB* (via ApoB reduction)	Ever-smokers	0.241	−0.079	−0.216	8.1
*APOB* (via TG reduction)	Ever-smokers	0.642	−0.079	−0.486	9.5
*APOC3* (via ApoB reduction)	LUAD	0.716	−0.069	−0.513	8.7

LLD, lipid-lowering drugs; ApoB, apolipoprotein B; TG, triglycerides; LUAD, lung adenocarcinoma.

## 4 Discussion

In this drug-target MR analysis, we assessed the effects of LLDs on lung cancer risk via six LLD targets (*APOB*, *APOC3*, *HMGCR*, *LPL*, *NPC1L1*, and *PCSK9*), comprising 12 lipid-lowering pathways. Our findings revealed that *APOB* inhibition decreased the lung cancer risk in ever-smokers and SCLC. *APOC3* inhibition decreased the LUAD risk but increased the SCLC risk. Additionally, *HMGCR* inhibition increased the risk of SCLC, whereas the *LPL* agonist decreased the risk of SCLC.

We delved deeper into the mediating mechanisms involving sphingolipid metabolites within these processes. Our investigation revealed that inhibiting *APOB*, *APOC3*, and *HMGCR* increased plasma neutral ceramidase levels. Notably, we observed that neutral ceramidase played a protective role in various forms of lung cancer, including overall lung cancer, lung cancer in ever-smokers, LUAD, and LUSC. These findings highlight the potential therapeutic significance of neutral ceramidase as a mitigating factor in the development and progression of various types of lung cancer. In addition, we found that *APOB* and *PCSK9* inhibition led to a reduction in plasma ceramide levels, while the *LPL* agonist increased plasma ceramide and S1P levels. Nonetheless, no substantiated evidence has emerged to establish a causal link between ceramides and the risk of lung cancer.

Several MR studies have explored the potential preventive effects of LLDs on malignancies (Carter et al., 2020; Liu et al., 2021a; Min et al., 2023); however, these investigations have primarily focused on statins and have not included stratified analysis of different subtypes of lung cancer. In contrast to previous MR studies, our study expands the range of LLD targets under consideration. In our positive control analysis, we observed that *ANGPTL3* inhibition did not achieve statistical significance. This finding aligns with the results reported by Wang et al. (2021). We made the decision not to conduct further analysis for *ANGPTL3* inhibition in subsequent MR analysis.

In contrast to previous MR analysis of the effects of statins on lung cancer risk, our study reveals novel findings that *HMGCR* inhibition increases the risk of SCLC, as does *APOC3* inhibition. This aligns with the findings reported by Vinogradova et al., where the link between statins and heightened lung cancer risk persisted even after adjusting for cardiovascular factors. However, their investigation lacked a subgroup analysis focusing on lung cancer types (Vinogradova et al., 2011). Moreover, evidence hints at the potential for long-term statin use to heighten cancer risk in women (Friedman et al., 2008). The mechanistic underpinnings behind the potential of statins in increasing SCLC risk remain elusive. We posited that this effect might stem from off-target consequences associated with prolonged statin use (Liu et al., 2021b; Jiang et al., 2023).

Immunohistochemical assessments of lung cancer tissue samples have unveiled lower protein expression of *APOC3* in SCLC compared to normal lung tissue. This discrepancy might propose a pathway through which *APOC3* inhibitors heighten the vulnerability to SCLC (Shi et al., 2016). Furthermore, an investigation under the UK’s Early Access to Medicines Scheme reported instances of lung cancer metastasis after the prolonged utilization of *APOC3* inhibitors (volanesorsen) (Jones et al., 2023). Our findings strongly advocate for heightened vigilance concerning the potential for *APOC3* inhibitors to increase lung cancer risk.

While alipogene tiparvovec acts as an *LPL* agonist, its limited market presence and high cost (Senior, 2017) have left the landscape of observational inquiries into its long-term cancer risk ambiguous. Nonetheless, our study propounds a shielding effect of *LPL* agonists against SCLC susceptibility. Moreover, Cerne et al. (2007) reported diminished *LPL* gene expression within lung cancer tissues as opposed to normal tissues, potentially reinforcing the notion of *LPL* agonists mitigating lung cancer risk.

The observed causal relationships between *APOB* inhibition and reduced lung cancer risk in ever-smokers and LUAD risk are particularly noteworthy, as neutral ceramidase plays a significant mediating role in these relationships. In recent years, there has been increasing research into the role and mechanisms of neutral ceramidase in malignancies (Coant and Hannun, 2019). Preclinical studies have demonstrated that inhibiting neutral ceramidase can prevent the occurrence and progression of colon cancer (Garcia-Barros et al., 2016), whereas inhibiting acid ceramidase can inhibit the proliferation of non-small-cell lung cancer and enhance its sensitivity to cisplatin (Yildiz-Ozer et al., 2018; White-Gilbertson et al., 2019). However, observational studies have presented contrasting findings, with high levels of acid ceramidase being associated with improved prognosis in breast and ovarian cancers (Ruckhaberle et al., 2009; Hanker et al., 2013; Sanger et al., 2015). The conflicting results from clinical observational studies and preclinical studies suggest that ceramidases may have pleiotropic effects on the occurrence, treatment, and prognosis of cancers. These findings highlight the complex and multifaceted nature of ceramidases in cancer-related processes. In our MR analysis, we identified a causal relationship between increased neutral ceramidase and a reduced risk of lung cancer in ever-smokers. This suggests that neutral ceramidase may play a critical role in the development of smoking-induced lung cancer, with *APOB* inhibition potentially acting as a protective factor. Further research is warranted to elucidate the precise mechanisms underlying their effects and to reconcile the disparities observed among different types of cancer.

Ceramidases play essential regulatory roles in the onset and advancement of various cancer forms (Sanger et al., 2015; Garcia-Barros et al., 2016) and are pivotal in sphingolipid metabolism for converting ceramides into sphingosine, S1P, and fatty acids (Parveen et al., 2019). The sphingolipid metabolism pathway proves indispensable in the metastatic process of lung cancer (Pyne et al., 2018; Coant and Hannun, 2019); its potential as a novel therapeutic target holds significant promise for augmenting the efficacy of tumor treatments (Vijayan et al., 2019), and animal experiments have demonstrated that *NPC1L1* inhibition can modulate sphingolipid metabolism (Yamanashi et al., 2020). Although observational studies have indicated an association between statin use and reduced plasma ceramide levels, there is limited information on the impact on ceramidase levels.

In alignment with the findings of a previous study, our study identifies increased plasma ceramide levels as a contributing factor to heightened lung cancer risk among non-smokers, albeit with nominal statistical significance (Alberg et al., 2013). Preclinical studies have shown that exposure to cigarette smoke leads to increased ceramide levels in the lung tissue of mice (Filosto et al., 2011; Goldkorn et al., 2014; Lavrynenko et al., 2020); the contribution of ceramides to the heightened risk of smoking-related lung cancer necessitates prospective investigations for conclusive evidence. Consistent with previous observational studies, we found that *PCSK9* inhibition reduced plasma ceramide levels (Tarasov et al., 2014), and *APOB* inhibition had the same effect. In addition, our results revealed that the inhibition of *LPL* could increase plasma S1P levels. However, owing to the non-convergence of the IVs for S1P in the lung cancer GWAS dataset, an analysis of the association between S1P and lung cancer risk was precluded. Notably, Alberg et al. (2013) observed an increased risk of lung cancer with increased S1P levels.

The strength of our study resides in its augmentation of LLD targets and lipid-lowering pathways. It amplifies the scrutiny of distinct lung cancer subgroups. More importantly, we delve into the mediating effect of sphingolipid metabolites, thus elucidating the intricate mechanism underpinning how LLD influences lung cancer risk. However, it is important to acknowledge the limitations of our study. First, MR analyses could not be performed on two of the target genes of LLD: *ANGPTL3* and *PPARA*. This limitation arises from the inability of IVs to establish positive control associations or the unavailability of suitable genetic variants. Furthermore, given the intricate pharmacological mechanisms underlying these drugs, our MR analysis was unable to assess their potential off-target effects. Second, IVs employed as proxies for lifetime exposure to LLD may be limited in their ability to capture the effects of short-term drug exposure. Additionally, our study is constrained to examining causal directions and does not enable a precise estimation of dosage effects or the cumulative impact of multiple medications. Third, although the F-statistic for the genetic variants exceeds 10, indicating a low probability of weak instrument bias, statistical power is constrained due to the limited availability of only two SNPs as IVs for acidic and neutral ceramidases (Larsson et al., 2019). Moreover, the relatively small sample size utilized for the assessment of sphingolipids raises concerns regarding potential selection bias. As a result, the interpretation of our findings should be exercised with caution. Fourth, despite sensitivity analyses not yielding statistically significant outcomes, it remains essential to acknowledge the potential impact of confounding variables and horizontal pleiotropy, given the possibility of SNPs residing in a state of weak linkage disequilibrium (*r* ^2^ < 0.30). Fifth, the absence of GWAS summary statistics for sphingolipids and lung cancer across diverse populations precludes validation. MR studies cannot substitute randomized controlled trials, and further clinical and pharmacoepidemiological studies are essential for triangulating. Sixth, the study population predominantly comprised individuals of European ancestry, which may limit the generalizability of our findings to other racial and ethnic groups.

## 5 Conclusion

We elucidated the intricate interplay between LLDs, sphingolipid metabolites, and lung cancer risk. Associations of *APOB*, *APOC3*, and *HMGCR* inhibition and the *LPL* agonist with distinct lung cancer risks underscore the multifaceted nature of these relationships. The observed mediation effects highlight the considerable influence of neutral ceramidase on lung cancer risk reduction achieved by *APOB* and *APOC3* inhibition.

## Data Availability

The datasets presented in this study can be found in online repositories. The names of the repository/repositories and accession number(s) can be found in the article/Supplementary Material.
